# Alpha2-Antiplasmin Limits Fibrinolysis by Tenecteplase and Enhances Brain Injury After Reperfusion in Ischemic Stroke

**DOI:** 10.3390/ijms27156558

**Published:** 2026-07-23

**Authors:** Satish Singh, Sofiyan Saleem, Ryan D. Sullivan, Guy L. Reed

**Affiliations:** 1 Translational Cardiovascular Research Center, Department of Medicine, University of Arizona College of Medicine, Phoenix, AZ 85004, USAssaleem1@arizona.edu (S.S.); ryansullivan@arizona.edu (R.D.S.); 2 CSIR—Institute of Himalayan Bioresource Technology, Palampur 176061, India

**Keywords:** tenecteplase, alpha2-antiplasmin, fibrinolysis, clot lysis, ischemic stroke, thrombolytic therapy

## Abstract

A bioengineered version of recombinant tissue plasminogen activator, i.e., tenecteplase (TNK-tPA), was designed to have a longer half-life, resistance to plasminogen activator inhibitor-1, and fibrin-targeted plasminogen activation. By comparison to tPA, clinical trials suggest that TNK-tPA may be less susceptible to the effects of alpha2-antiplasmin (α2AP), the primary inhibitor of thrombus dissolution. However, preclinical studies are limited, and whether α2AP affects TNK-tPA’s fibrinolytic activity or efficacy in experimental ischemic stroke is unknown. We examined the effects of TNK-tPA and α2AP on the dissolution of human plasma clots (in vitro) and experimental ischemic brain injury from stroke induced by transient middle cerebral artery ischemia. TNK-tPA induced a dose-dependent increase in plasma clot dissolution; inhibition of α2AP with a specific monoclonal antibody caused a synergistic increase in TNK-tPA-mediated clot dissolution. In experimental ischemic stroke with ischemia and reperfusion, TNK-tPA treatment of α2AP^−/−^ mice significantly reduced ischemic infarct volume, brain swelling, brain hemorrhage, and neurobehavioral disability vs. TNK-tPA-treated α2AP^+/+^ (C57BL/6 background) mice with normal α2AP levels (*p* < 0.05 to *p* < 0.0001). α2AP impairs the dissolution of human clots in vitro by TNK-tPA and significantly exacerbates ischemic brain injury, swelling, hemorrhage, and neurobehavioral disability after experimental stroke, even with reperfusion. Targeting α2AP may improve the efficacy of TNK-tPA and reduce hemorrhagic complications.

## 1. Introduction

Tenecteplase (TNK-tPA) is a genetically engineered version of tissue plasminogen activator (tPA) [1]. Specific mutations increase its half-life due to altered glycosylation and reduce its neutralization by plasminogen activator inhibitor-1 (PAI-1) [1]. TNK-tPA (0.25 mg/kg) is now preferred clinically due to its faster and easier bolus administration vs. the standard dose (0.9 mg/kg) tPA, which is given by continuous infusion [1,2,3,4]. Nevertheless, TNK-tPA has not been shown to be superior to tPA for overall mortality, disability, and hemorrhage in ischemic stroke patients in ongoing trials [2,4,5]. By comparison to tPA, preclinical studies of TNK-tPA are limited, and the factors that alter fibrinolytic potential and efficacy in ischemic stroke are not fully understood.

Plasminogen activation therapy by tPA or TNK-tPA generates plasmin to accelerate fibrinolysis or dissolution of obstructing blood clots [6,7,8,9] (Supplementary Figure S1). Alpha2-antiplasmin (α2AP) is the primary inhibitor of plasmin and regulates fibrinolysis [10,11,12,13]. It is synthesized in the liver, released into blood, and cross-linked to fibrin by factor XIIIa, strengthening clots against plasmin-mediated dissolution. Genetic and acquired deficiency influences the circulating levels of α2AP [11,14], which may predispose individuals to differing fibrinolytic potential and response to thrombolytic therapy. Specifically, in ischemic stroke patients, lower pretreatment plasma levels of α2AP are associated with greater recanalization with thrombolysis by tPA [15]. Higher serum α2AP levels in patients on admission are associated with increased resistance against thrombolysis by intravenous tPA and poorer functional outcomes [16]. In experimental thromboembolic ischemic stroke, we have previously shown that endogenous α2AP has deleterious effects on thrombus dissolution and disease outcomes [17,18]. Also, these studies noted that increased α2AP levels reduce the response to tPA treatment in thromboembolic stroke. In ischemic stroke patients, increased α2AP levels also affect reperfusion by tPA, though the effect of α2AP on TNK-tPA has not been examined [15]. It is reasonable to expect that α2AP also affects clot dissolution and ischemic stroke outcomes by TNK-tPA treatment [15,17,18]. However, by comparison to tPA, TNK-tPA is reported to have greater fibrin-dependent plasminogen activation, and clinical studies suggest that TNK-tPA is less susceptible to inhibition by physiological inhibitors of fibrinolysis, e.g., α2AP [19]. Preclinical studies are limited, and the precise impact of α2AP on TNK-tPA’s fibrinolytic activity and efficacy in experimental ischemic stroke is translationally important but remains unknown. We hypothesized that despite its bioengineered fibrin specificity, TNK-tPA’s effects remain critically limited by endogenous α2AP and that neutralizing this inhibitor would rescue both fibrinolytic and neurological outcomes.

To address this knowledge gap, we systematically examined the effects of α2AP on TNK-tPA-induced human plasma clot dissolution in vitro. We also determined whether endogenous α2AP affects ischemic brain injury during thrombolytic treatment by TNK-tPA in experimental ischemic stroke. This study directly addresses the central role of fibrinolytic factors in disease by establishing α2AP as a primary bottleneck governing TNK-tPA-mediated clot lysis. By pairing real-time kinetic assays of plasma clot turbidity with an in vivo model of ischemic stroke reperfusion injury, we uncover a novel, synergistic interplay between TNK-tPA and α2AP inactivation (α2AP-I).

## 2. Results and Discussion

### 2.1. Synergistic Effects of TNK-tPA and α2AP-I on Human Plasma Clot Lysis

**Figure 1:**
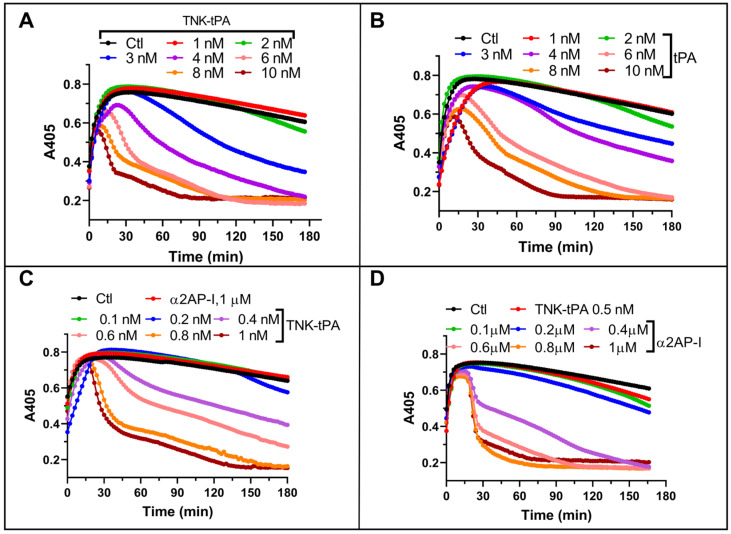
**Kinetics of human plasma clot lysis by different agents.** Plasma clot formation (increase in absorbance) and lysis (decrease in absorbance) were examined by the turbidity method, measuring absorbance at 405 nm. Graphs (**A**,**B**) show dose-dependent effects of TNK-tPA and tPA on plasma clot lysis, respectively. The reaction buffer Tris was used as a negative control (Ctl). Graph (**C**) shows plasma clot lysis with varying sub-nanomolar concentrations of TNK-tPA in the presence of a fixed concentration of α2AP-I (1 µM). Graph (**D**) shows the effects of varying concentrations of α2AP-I at a fixed sub-nanomolar concentration of TNK-tPA, i.e., 0.5 nM. For each agent or control, one representative graph from 6 to 8 repeated experiments (in duplicates) is shown.

We first compared the dose-dependent effects of TNK-tPA to tPA on plasma clot lysis. TNK-tPA showed a dose-dependent (1–10 nM) increase in plasma clot lysis comparable to tPA (Figure 1A,B). In this assay, by comparison to controls (no agent), a threshold dose of 1–3 nM TNK-tPA was required for a noticeable increase in clot lysis, whereas 8–10 nM TNK-tPA achieved nearly the maximum rate of lysis (Figure 1A).

**Figure 2:**
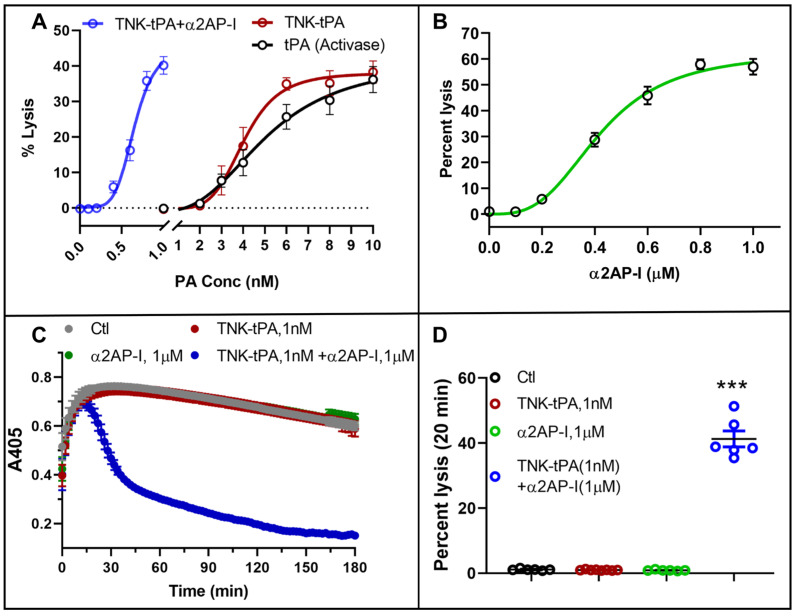
**Quantitative measurement of dose-related effects of agents on plasma clot lysis. Panels** (**A**,**B**): Graph (**A**) shows the comparative measurement of dose-related effects of TNK-tPA (1–10 nM), tPA (1–10 nM), and TNK-tPA (0.1–1.0 nM) + α2AP-I (1 µM; fixed dose) in the plasma clot lysis assay. PA denotes plasminogen activator. Graph (**B**) shows the dose-related effects of α2AP-I on plasma clot lysis with a fixed low dose of TNK-tPA (0.5 nM) over time (t = 20 min). Panels (**C**,**D**): Panel (**C**) shows the plasma clot lysis graphs for low-dose TNK-tPA with or without α2AP-I. Panel (**D**) shows the quantitative measurement of the synergistic effects of low-dose TNK-tPA with α2AP-I. The concentrations are mentioned in the graph. The reaction buffer Tris was used as a negative control (Ctl). The percent lysis (% decrease in absorbance from t = 0 to t = 20 min) values were derived from the plasma clot lysis graphs shown in panel (**C**). Clot lysis experiments were performed in duplicate and repeated 6–8 times. Data are represented as mean ± SEM. Synergistic interaction between TNK-tPA (1 nM) and α2AP-I (1 μM) was evaluated using the Bliss independence multiplicative model. The comparisons were done using a one-sample *t*-test, and group differences were assessed by one-way ANOVA. *** *p* < 0.001 vs. control.

The EC_50_ (i.e., effective concentration to achieve half of the maximum clot lysis rate) of TNK-tPA was 4.04 ± 0.29 nM (Figure 2). TNK-tPA showed comparable clot lysis ability to tPA with a similar EC_50_ (5.10 ± 0.96 nM) (Figure 2).

To investigate the specific modulatory effects of α2AP on TNK-tPA-mediated plasma clot lysis, the assays were performed utilizing 10-fold reduced doses of TNK-tPA (0.1–1 nM) combined with α2AP-I [20] (Figure 1C and Figure 2A). α2AP-I was used at equimolar doses to neutralize the α2AP in plasma (i.e., 1 µM) [10] in combination with sub-nanomolar doses of TNK-tPA to mimic the physiological concentration of plasminogen activator (tPA). Low-dose TNK-tPA (0.1–1 nM) showed a dose-dependent increase in clot lysis in the presence of α2AP-I (1 µM) (Figure 1C and Figure 2A). α2AP-I reduced the EC_50_ for TNK-tPA-mediated clot lysis six-fold (i.e., 4.04 ± 0.29 nM without α2AP-I vs. 0.65 ± 0.04 nM with α2AP-I) (Figure 1C and Figure 2A), demonstrating a dramatic leftward shift in therapeutic potency.

We then evaluated the dose-dependent effects of α2AP-I (0.1–1 µM) in the presence of a fixed, low dose of TNK-tPA (0.5 nM) (Figure 1D and Figure 2B). The EC_50_ value for the α2AP-I in this assay was 0.42 ± 0.025 µM (i.e., 420 nM). To further analyze the mathematical nature of this enhancement, we quantified the individual versus combinatorial effects of low-dose TNK-tPA and α2AP-I on the absolute percentage of clot lysis (Figure 2C,D). In comparison with the negligible clot lysis by TNK-tPA (1 nM) or α2AP-I (1 µM) alone, the combination of TNK-tPA + α2AP-I caused an approximately 37-fold increase in clot lysis (mean ± SD: 41.28 ± 6.01 vs. 1.12 ± 0.26; *p* < 0.001), yielding a Bliss synergy score of 36.30 and confirming a statistically significant synergistic interaction (t = 16.51, *p* < 0.001) (Figure 2D). These data establish that α2AP was a major deterrent to TNK-tPA-mediated plasma clot lysis.

### 2.2. Fibrinolysis Is Restricted by Physiological α2AP Through Plasmin Inhibition

α2AP is a known inhibitor of the fibrinolytic enzyme plasmin and thus fibrinolysis [6,11]. Because TNK-tPA and tPA displayed similar kinetic profiles in initial assays, we utilized tPA as a representative plasminogen activator to examine the mechanism of the synergistic effects of α2AP-I on plasminogen activator-mediated plasma clot lysis using our dual activity assay.

**Figure 3:**
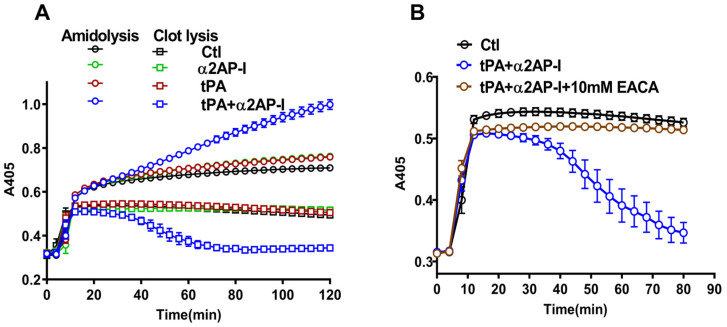
**Simultaneous measurement of clot lysis and amidolysis in a plasma clot lysis assay.** The effects of tPA (0.8 nM), α2AP-I (1.6 µM), or tPA (0.8 nM) + α2AP-I (1.6 µM) were measured in a dual activity assay. (**A**) The graphs with colored square symbols with A405 below 0.6 represent the clot turbidity measurements in duplicate wells; the graphs with colored circle symbols with A405 above 0.6 represent amidolysis + turbidity measurements. The graphs show that the effects of tPA + α2AP-I on plasma clot lysis correspond to plasmin’s catalytic activity (**B**). The graph shows the clot turbidity measurements at 405 nm for the reactions containing tPA (0.8 nM); α2AP-I (1.6 µM); tPA (0.8 nM) + α2AP-I (1.6 µM); or tPA (0.8 nM) + α2AP-I (1.6 µM) + 10 mM EACA. Experiments were performed in duplicate each time (n = 5–8).

Figure 3 shows that at the given concentrations of tPA and α2AP-I, neither agent was capable of inducing noticeable fibrinolysis, and the corresponding amidolytic activity of plasmin was negligible. However, a combination of tPA with α2AP-I dramatically increased the clot lysis, which was also associated with a marked increase in plasmin’s amidolytic activity. In an independent experiment, we found that inhibiting plasmin’s activity by ε-aminocaproic acid (EACA, 10 mM) reversed the synergistic effects of tPA + α2AP-I on plasma clot lysis (Figure 3B).

These results show that the plasma clot lysis ability of TNK-tPA or tPA is drastically reduced by the presence of endogenous levels of α2AP. Fibrinolysis can be accelerated with minimum plasminogen activation when the inhibitory effects of physiological α2AP are removed. α2AP forms a rapid 1:1 stoichiometric complex with plasmin [6,11]. Neutralization of α2AP allows smaller amounts of plasmin to achieve greater fibrinolysis, allowing fibrin-generated plasmin, in particular, to have a longer catalytic half-life. Equivalent amounts of fibrinolysis may be achieved by high-dose TNK-tPA, generating large amounts of plasmin. However, high doses of TNK-tPA and high plasmin levels have off-target, toxic effects that have limited their safety in clinical trials [21].

To mathematically characterize the nature of interactions for the combined effect of low-dose TNK-tPA and α2AP-I, we utilized the Bliss independence multiplicative model [22]. This method was chosen specifically to evaluate synergistic effects of combinations of drugs that operate through distinct non-overlapping targets (i.e., plasminogen activation and plasmin inhibition). Synergistic interactions between TNK-tPA (1 nM) and α2AP-I (1 μM) were evaluated, and the comparisons were done using a one-sample *t*-test, and group differences were assessed by one-way ANOVA.

### 2.3. α2AP Worsens Cerebral Infarction, Brain Swelling, Hemorrhage, and Neurobehavioral Function During TNK-tPA Treatment in Ischemic Stroke

**Figure 4:**
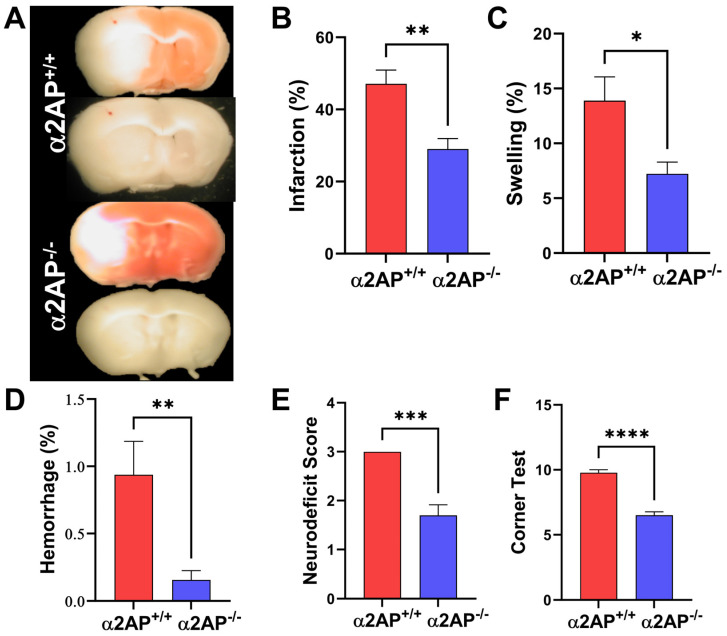
**α2AP affects outcomes after TNK-tPA treatment of experimental ischemic stroke with reperfusion.** Bar graphs compare ischemic stroke outcomes in mice with normal α2AP levels (α2AP^+/+^) and α2AP-deficient mice (α2AP^−/−^). (**A**) Examples of unstained and TTC-stained 2 mm sections of the brain for the measurement of hemorrhage (arrow) and cerebral infarction (unstained pale area). (**B**) Quantitative measurement of cerebral infarct volume (%), (**C**) brain swelling and (**D**) brain hemorrhage. Neurobehavioral assessment of functional impairment by the (**E**) neurological deficit score (neurodeficit score) and (**F**) Corner’s test. * *p* < 0.05, ** *p* < 0.01, *** *p* < 0.001, **** *p* < 0.0001 by the Mann–Whitney U-test, which was used for all comparisons. The average weight of (α2AP^+/+^) and α2AP-deficient mice (α2AP^−/−^) was 24.6 ± 2.2 g vs. 25 ± 1.8 g respectively; the average age was 95 ± 15 vs. 75 ± 10 days, mean ± SE shown. Experiments were performed in a randomized and blinded fashion, n = 20, with 10 male mice in each group.

To determine whether the in vitro synergistic effects of α2AP-I and TNK-tPA translate to improved outcomes in vivo, we utilized a filament-occlusion transient ischemic stroke model in congenic α2AP^+/+^ vs. α2AP^−/−^ mice [17,18,23]. Brain ischemia was induced by filament occlusion of the middle cerebral artery (MCA) for 2 h, followed by reperfusion and treatment [24,25]. When compared to TNK-tPA-treated wild-type (α2AP^+/+^) mice, TNK-tPA-treated α2AP^−/−^ mice showed a 53% reduction in mean ischemic infarct volume (*p* = 0.0041, d = 1.5738, 95% CI: −32.55 to −6.684) (Figure 4A,B).

Similarly, mean brain swelling was reduced 48% (*p* = 0.0350; d = 0.7167, 95% CI: −12.92 to −0.09666; Figure 4C), and brain hemorrhage was decreased by 83% (*p* = 0.0034; d = 5.12, 95% CI: −1.001 to −0.2355; Figure 4A,D) in TNK-tPA-treated α2AP^−/−^ vs. α2AP^+/+^ mice. Genetic deficiency of α2AP significantly reduced neurobehavioral disability during TNK-tPA treatment at 24 h post-stroke induction. TNK-tPA-treated α2AP^−/−^ mice had significantly improved neurobehavioral Bederson scores vs. TNK-tPA-treated α2AP^+/+^ mice (*p* = 0.001; d = 2.6473, 95% CI: −2.000 to −1.000; Figure 4E). Similarly, sensorimotor dysfunction as measured by the corner test was also significantly reduced in α2AP^−/−^ vs. α2AP^+/+^ mice with TNK-tPA treatment (*p* = 0.0001; d = 4.2622, 95% CI: −4.000 to −3.000; Figure 4F). These data show that endogenous α2AP increases cerebral infarction, brain swelling, hemorrhage, and neurobehavioral impairment in TNK-tPA-treated, reperfused mice with ischemic stroke.

While clinical studies have suggested that TNK-tPA’s genetic modifications, including increased fibrin specificity and localized plasminogen activation, render it less susceptible to endogenous inhibitors [19,26], these experimental data show that α2AP is a potent regulator of its fibrinolytic potential. The fibrinolytic potency of TNK-tPA for dissolving human clots was reduced ~6-fold by α2AP, and the combination of low-dose TNK-tPA and α2AP-I was markedly synergistic. The 37-fold increase in clot lysis observed with the combination, supported by a Bliss synergy score of 36.30, highlights that α2AP is not merely a modulator but a primary bottleneck for TNK-tPA efficacy. In vivo studies showed that α2AP also impaired the ability of TNK-tPA to reduce ischemic brain injury in experimental ischemic stroke with reperfusion, even when TNK-tPA was given alone at doses equivalent to those shown to achieve maximal fibrinolytic reperfusion in clinical studies [26]. At these doses, TNK-tPA in α2AP-deficient mice significantly reduced ischemic brain injury, hemorrhage, edema, and neurological disability. Although in vivo thrombolysis was not examined, these experiments showed that fibrinolysis of human clots was enhanced by both TNK-tPA and tPA in vitro when α2AP is inhibited. Our previous in vivo studies have shown that fibrinolysis of an MCA thromboembolus by tPA is enhanced, and ischemic brain injury and disability are reduced by α2AP inactivation [17,18]. These deleterious effects of α2AP on TNK-tPA activity in experimental ischemia–reperfusion stroke are consistent with the harmful effects noted for α2AP during experimental thromboembolic ischemic stroke. However, the neuroprotective effect in α2AP^−/−^ mice observed in this filament-induced stroke model does not appear to be due to enhanced thrombolysis but rather may be due to reduced thromboinflammation with preservation of microvascular patency and blood–brain barrier protection [18,27,28].

Completely unchecked plasmin activity creates a systemic lytic state that carries a potential hemorrhagic risk through degradation of fibrinogen and clotting factors and induction of blood–brain barrier breakdown [21]. However, α2AP deficiency itself does not cause a systemic fibrinolytic state in humans or mice because the activity of plasmin is also regulated by other inhibitors such as alpha2-macroglobulin [6,12,23]. Our data show that in α2AP^−/−^ mice receiving TNK-tPA, the absence of α2AP reduced brain hemorrhage by 83% (*p* < 0.01; Figure 4A,D). Previous experimental studies with tPA and α2AP deficiency have also shown reduced brain hemorrhage in mice [17,18]. The 83% reduction in hemorrhage in α2AP^−/−^ mice suggests that the function of α2AP extends beyond fibrinolysis, which may drive microvascular instability, thromboinflammation, and blood–brain barrier breakdown. These in vivo studies used mice with constitutive genetic deficiency of α2AP, whereas the in vitro synergy in human plasma clot lysis assays was produced by acute antibody-mediated inhibition with TS23, which specifically inactivates human α2AP [10]. This distinction favors clinical translation, as acute, titratable inhibition at the time of thrombolysis would transiently lower the α2AP threshold. The use of two independent approaches, i.e., genetic deficiency in vivo and antibody inhibition in vitro, supports the robustness of α2AP as a target. We note, however, that we did not examine whether mouse sex alters outcomes, nor did we perform in vivo dose titration, although that has been done for combinations of tPA and α2AP inhibitors [17,18]. We did not examine the effects of α2AP deficiency/inhibition on long-term functional outcomes beyond 24 h, but previous experimental studies showed that α2AP inhibition alone was associated with longer survival after stroke [17,18].

Taken together, these results provide important insights into how α2AP affects in vitro fibrinolysis and the therapeutic potential of TNK-tPA even with reperfusion. Since α2AP reduces plasma clot lysis by TNK-tPA and enhances ischemic brain injury, targeting α2AP may prove useful during TNK-tPA therapy for ischemic stroke.

## 3. Materials and Methods

### 3.1. Plasma Clot Lysis Assay

Citrated human plasma (Lampire Biological Laboratories, Pipersville, PA, USA) clot lysis was performed in Tris buffer (50 mM Tris, 125 mM NaCl, pH 7.4) by the clot turbidity method at 37 °C [29]. Plasma was diluted to 70% in Tris buffer, and 10 mM CaCl_2_ was added to the reaction mixture. tPA (Activase^®^-Cathflo^®^; Genentech, South San Francisco, CA, USA), TNK-tPA (Metalyse^®^; Boehringer Ingelheim, Ingelheim am Rhein, Germany), or α2AP-I agent was either added to the reaction mixture (fixed concentration) or the well of a microtiter plate (varying concentration) containing thrombin (0.3 U/mL; Sigma-Aldrich, St. Louis, MO, USA). α2AP-I was achieved using TS23, a monoclonal antibody that specifically inactivates human α2AP [20]. The reaction mixture was then added to the wells, and the reaction was monitored at 405 nm at 37 °C using a BioTek Synergy HT plate reader (Winooski, VT, USA) for 3 h. Nonlinear regression analysis and curve fitting were performed for the graphs of dose-related effects of different agents on percent clot lysis using a sigmoidal dose–response equation in GraphPad Prism 8.0 (San Diego, CA, USA) to calculate the EC_50_ value (n = 6–8 experiments). The EC_50_ was defined as the effective concentration of the agent at which 50% of the maximum lysis rate is achieved in the given time interval. For the calculation of clot lysis, the maximum absorbance (A405) or maximum turbidity was used as 100%, i.e., completion of clot formation in each reaction, and the percent decrease in absorbance (from maximum turbidity, i.e., t = 0) was calculated as percent lysis over time (e.g., t = 20 min). The percent lysis at a given time point in the propagation phase of the reaction was plotted against the varying concentration of the agent, i.e., TNK-tPA, tPA, or α2AP-I.

### 3.2. Measurement of Fibrinolysis and Amidolysis by Plasmin During Plasma Clot Lysis

A novel dual-activity plasma clot lysis assay was developed. This assay simultaneously measures plasmin’s fibrinolytic activity via clot turbidity and its amidolytic activity via p-nitroanilide release from the chromogenic substrate S2251 (MyBiosource Inc., San Diego, CA, USA). Diluted, citrated multidonor human plasma (50% in 50 mM Tris, 125 mM NaCl, pH 7.4) was incubated with TS23 antibody (1.6 µM) for 10 min at 37 °C. An equal volume of buffer was added to the control plasma. 0.5 mM S2251 or buffer and 16 mM CaCl_2_ were added to the tubes, and the reaction mix was immediately added to the wells containing Dade Innovin (Siemens, Munich, Germany; prepared as per manufacturer’s guidelines; used 2 µL of 1000× dilution) and/or fibrinolytic agent. The effects of tPA (0.8 nM), α2AP-I (1.6 µM), or tPA (0.8 nM) + α2AP-I (1.6 µM) were measured. Each reaction (100 µL) was set up in duplicate wells, and the parallel controls in the adjacent wells (in duplicate) were also used, in which S2251 was not added. The reactions were monitored at 405 nm at 37 °C continuously in the microplate reader.

### 3.3. Ischemic Stroke by Transient Occlusion of the MCA

Animal experiments were performed under the approved protocols by the ‘Institutional Animal Care and Use Committee’ of the University of Arizona, following the ARRIVE guidelines [30,31]. The mice were housed in ventilated cages in the animal house of the university with a temperature of ~22 °C and a light/dark cycle of 12 h each. Ischemic stroke in adult (8–12 weeks of age) α2AP^+/+^ and α2AP^−/−^ mice (male) on C57BL/6 background (UC Davis, KOMP, CA, USA) was induced by filament-based transient occlusion of the MCA as previously described [24,25,32,33]. The mice were randomly assigned by an independent investigator to the experimenter (randomized in GraphPad Prism), who was blinded to the genotype for experimentation and quantification of the stroke outcomes. After completion of the full study, including measurements of outcomes and data analysis, the genotypes of the mouse groups were revealed to the experimenter. Surgery was performed under continuous anesthesia (1.5–2% isoflurane, with induction at 4%) (VetOne, Boise, ID, USA) delivered via a nose cone, and body temperature was maintained at 37 °C using a digitally controlled heating pad. Buprenorphine (SR, 1 mg/kg) was given as analgesia before surgery or when required during or post-surgery if the mice appeared to be in pain. A fiberoptic probe was affixed to the skull to monitor the relative cerebral blood flow of the affected area of the brain using laser-Doppler flowmetry (LDF, AD Instruments, Oxford Optronix, Oxford, UK). After a small incision in the neck area, the carotid artery bifurcation was exposed, and the common carotid artery was temporarily ligated. The MCA was occluded by the careful insertion of a silicone-coated Ethilon 7-0 nylon monofilament (Doccol Corp., Sharon, MA, USA). An incision was made in the external carotid artery stump, and the filament was introduced through the internal carotid artery to the origin of the MCA. An 80% drop in the blood flow measured by LDF was documented as a successful occlusion. The filament was left in the artery to create ischemia, the neck wound was sutured, and the animals were transferred to the temperature-regulated chamber. After 2 h, the mice were anesthetized to slowly remove the filament to allow reperfusion, a procedure that has been previously reported to induce reperfusion (measured by LDF) [24,34]. TNK-tPA was then given as a single intravenous bolus at a dose of 5 mg/kg through the jugular vein [35]. In rodents, a 10-fold higher dose than the human standard is typically required to match clearance rates, making 2.5 mg/kg, for example, equivalent to the clinically recommended human dose of TNK-tPA (0.25 mg/kg). We administered a dose of 5 mg/kg because it reliably induces intracerebral hemorrhage in experimental stroke models. By utilizing this hemorrhage-priming regimen, we aimed to rigorously evaluate the contribution of α2AP to severe bleeding complications associated with high-dose thrombolytic therapy. A similar dose of TNK-tPA (i.e., 4 mg/kg) was recently reported to be more effective in reducing cerebral infarction and brain hemorrhage in hyperglycemic mice during ischemic stroke [36]. The mice were allowed to recover from anesthesia after treatment and returned to the chamber for surgical recovery. To minimize confounders, only two surgeries per day were performed, and the mice were allowed to recover in properly labeled (numbers/letters from the randomization chart) individual cages. They were subjected to neurobehavioral tests at 24 h. After euthanasia at 24 h, the blood was collected by cardiac puncture for plasma preparation in 3.8% sodium citrate buffer. The mice were perfused with 0.9% saline (1.5–2 mL) through the heart, and the brain tissue was harvested immediately. All the experimental data were included for analysis unless they met the exclusion criteria set during pre-surgery experimental design, which included (a) if 80% occlusion could not be achieved as per LDF measurement, and (b) if the blinded experimenter noted a technical failure during the experiment. One α2AP^+/+^ mouse died during recovery prior to testing.

#### 3.3.1. Measurement of Ischemic Infarct Volume, Brain Swelling, and Hemorrhage

Immediately after extraction, the whole brain tissue was digitally photographed from both faces for the measurement of swelling [24,25]. The brain was sliced coronally into 2 mm thick sections to capture images from both faces for the measurement of hemorrhage. The sectioned tissue was stained with 2,3,5-triphenyltetrazolium chloride (TTC, 2%, Sigma) for the measurement of infarct volume [37]. Infarction volume was used as a primary outcome measure. Quantitative measurements of the area for brain swelling, hemorrhage, and cerebral infarction were performed on digital photographs by a blinded observer using the image analysis software Image-Pro Plus 6.2 (Rockville, MD, USA) [17,18,24]. The TTC-stained area from the ipsilateral (ischemic) and contralateral (non-ischemic) hemispheres from both faces was measured. For volume measurements, area was multiplied by slice thickness, i.e., 2 mm. Ischemic infarct volume (%) was quantified by using Swanson’s formula [38], i.e., infarct volume (%) = 100 × [(V_C_ − V_L_)/V_C_], where V_C_ = TTC-stained area in the control hemisphere × slice thickness, V_L_ = TTC-stained area in the infarct hemisphere × slice thickness [17,18,24]. The percent hemorrhage was quantified using the formula, i.e., hemorrhage (%) = 100 × (volume of hemorrhage (red color bleeds) in the infarcted hemisphere/volume of the control hemisphere) [17,24]. Similarly, the brain swelling was calculated by comparing the volume of the ischemic vs. non-ischemic hemisphere using the formula = 100 × (volume of the infarcted hemisphere–volume of the contralateral hemisphere)/volume of the contralateral hemisphere [17,24].

#### 3.3.2. Neurological Behavioral Outcomes

Neurological behavioral tests were performed by neurological deficit score (NDS) and corner test 24 h post-stroke before euthanasia [32,39]. NDS was quantified by the foot-fault test using Bederson scoring, defined as grade 0 (no deficit); grade 1 (failure to extend the left forepaw fully); grade 2 (circling to the left side); grade 3 (no spontaneous locomotor activity, with depressed level of consciousness). The corner test was performed as we have recently described [24,32]. The mice were allowed to move through artificially created corners by cardboard pieces. Unchallenged mice are capable of turning left and right equally, whereas mice with ischemic stroke tend to turn more toward the side of ischemic injury. The total number of right and left turns (out of a total of 10 turns) was counted and plotted in GraphPad Prism software.

### 3.4. Statistical Analysis

Statistical analysis of the data was performed in the GraphPad Prism 8.0 (San Diego, CA, USA) software. Data were expressed as mean ± SE. In vivo, data were tested for normality using the Shapiro–Wilk test. Group differences were analyzed using an unpaired *t*-test (parametric) or Mann–Whitney test (non-parametric) for two groups and one-way ANOVA for multiple groups. *p* < 0.05 was considered significant. Infarction volume was used as a primary outcome measure. Based on our previous studies [24,25], with a sample size of 10 mice in each group, the study had >82% power to detect a difference of 12% for infarction with an estimated standard deviation of 11.3%. The other key measures were estimated to have larger effect sizes, indicating a power > 80% for detecting differences.

## Data Availability

All data generated or analyzed during this study are included in this published article. Additional underlying source files and datasets are available from the corresponding author upon reasonable request.

## Supplementary Materials

The following supporting information can be downloaded at: https://www.mdpi.com/article/10.3390/ijms27156558/s1.

## Abbreviations

The following abbreviations are used in this manuscript:

TNK-tPA	Tenecteplase
tPA	Tissue plasminogen activator
α2AP	Alpha2-antiplasmin
α2AP-I	Alpha2-antiplasmin inactivation
PAI-1	Plasminogen activator inhibitor-1
EACA	ε-aminocaproic acid
MCA	Middle cerebral artery
LDF	Laser doppler flowmeter
TTC	2,3,5-triphenyltetrazolium chloride
NDS	Neurological deficit score
