# Supplementary material for: Alpha2-Antiplasmin Limits Fibrinolysis by Tenecteplase and Enhances Brain Injury After Reperfusion in Ischemic Stroke
Source: Int J Mol Sci. 2026 Jul 23;27(15):6558. doi: 10.3390/ijms27156558 (PMC13465430; doi:10.3390/ijms27156558)
Supplement: Supplementary file 1 [file ijms-27-06558-s001.zip › ijms-4389576-supplementary.pdf]

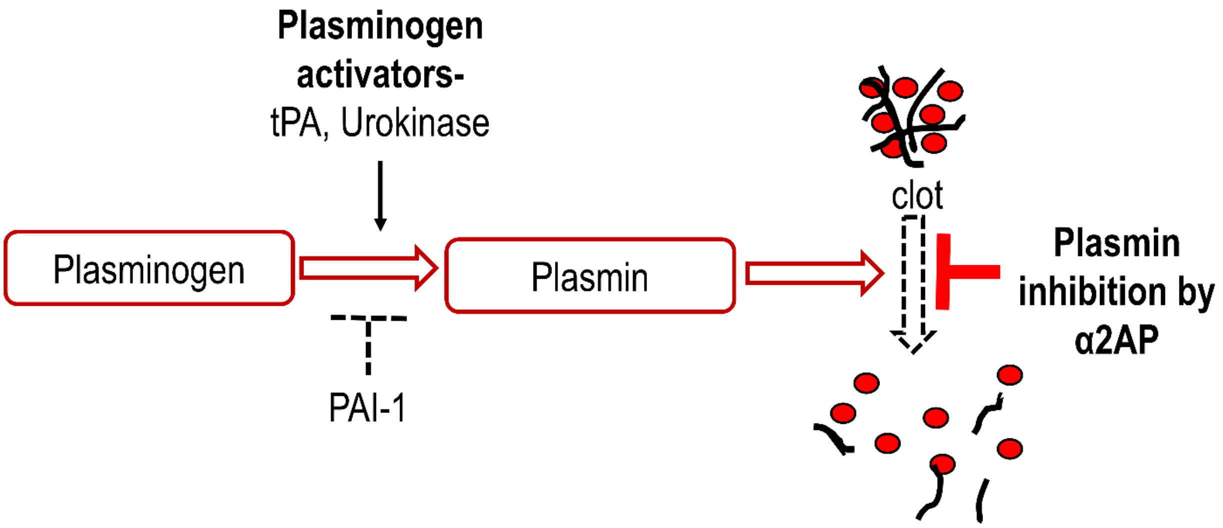

Supplementary Figure S1- Schematic representation of the fibrinolytic cascade and its primary regulators. Plasminogen activators, such as tissue plasminogen activator (t-PA), cleave the inactive zymogen plasminogen into active plasmin. Active plasmin then drives the dissolution of the fibrin clot into degradation products. This enzymatic pathway is tightly regulated by endogenous inhibitors: plasminogen activator inhibitor-1 (PAI-1) blocks the initial activation phase, and alpha2-antiplasmin (α2AP) acts as the primary down-stream check by directly binding and neutralizing active plasmin to arrest clot dissolution.
